# Bipolar clavicular fracture on ipsilateral reverse shoulder prosthesis: Case report

**DOI:** 10.1016/j.ijscr.2019.10.082

**Published:** 2019-11-03

**Authors:** Joseph Maalouly, Dany Aouad, Jamal Saade, Ghadi Abboud, Georges El Rassi

**Affiliations:** Department of Orthopedic Surgery and Traumatology, Saint Georges University Medical Center, Balamand University, P.O. Box 166378 Achrafieh, St Georges Street, Beirut, 1100 2807, Lebanon

**Keywords:** Bipolar, Clavicle, Fracture, Trauma, Reverse shoulder prosthesis

## Abstract

**Background:**

Bipolar clavicular fractures are extremely rare with no consensus on management. No previous cases of bipolar clavicular fracture on an ipsilateral reverse shoulder prosthesis have been reported.

**Case presentation:**

We report a case of a 78y.o female patient with left bipolar clavicular fracture on ipsilateral reverse shoulder prosthesis, associated with multiple ribs fracture, that was treated with open reduction and internal fixation using k-wires and endobutton for the lateral part, while as the medial part was fixed with plate and screws.

**Conclusions:**

Any patient with a clavicle fracture, with a history of sequential forces to the clavicle, a bipolar injury should always be suspected. Attention should be given if any implants are present on the ipsilateral side of the injury, which could affect surgical planning and outcome.

## Highlights

• Rare case of traumatic bipolar clavicular over an ipsilateral reverse shoulder prosthesis.
• Open reduction and internal fixation showed satisfactory bony alignement with intact reverse shoulder prosthesis.
• Post operative rehabilitation is of great importance, taking into consideration the deltoid musculature wish should be addressed for shoulder strengthening over prosthesis.

## 1 Background

Clavicle fractures are quite common, comprising 2.6–4% of all adult fractures [1,2]. However, segmental fractures, proximal part and midshaft or midshaft and distal part, are less common and bipolar fractures are the rarest. Due to their rarity, no standardized management consensus exists. We report the case of a 78y.o female patient with left bipolar clavicular fracture on ipsilateral reverse shoulder prosthesis that was treated with open reduction and internal fixation.

## 2 Case report

**Fig. 1:**
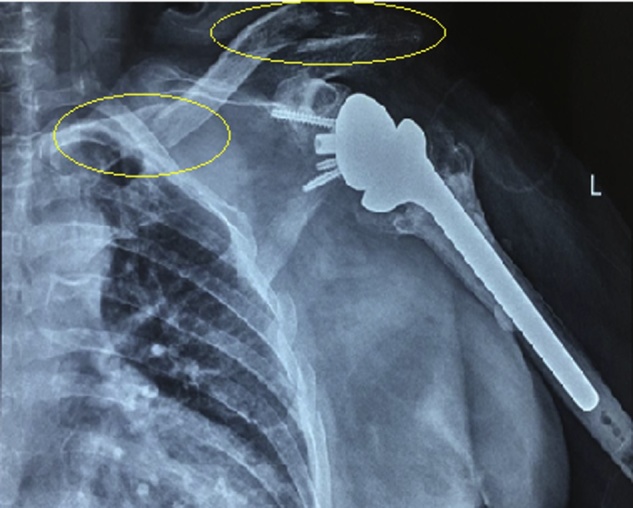
X-ray radiograph of the left clavicle and shoulder joint showing a proximal and distal clavicle fractures with intact reverse shoulder prosthesis in good alignment.

**Fig. 2:**
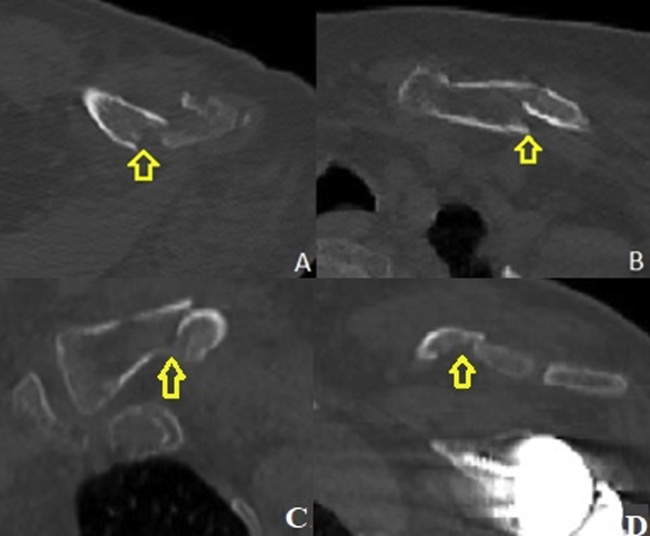
CT scan of the Left shoulder (A) axial cut showing distal displaced clavicle fracture (B) axial cut showing proximal displaced clavicle fracture (C) coronal cut showing proximal displaced clavicle fracture (D) coronal cut showing distal displaced clavicle fracture with intact glenoid component of previous reverse shoulder prosthesis.

**Fig. 3:**
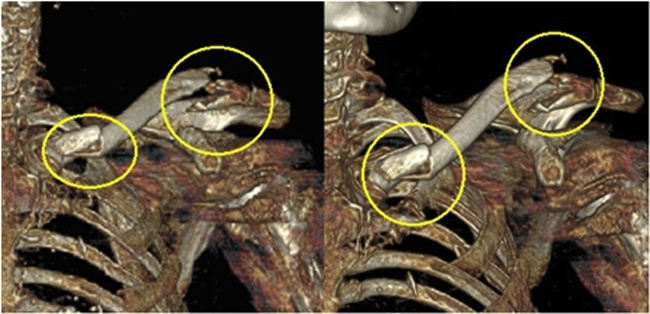
CT scan 3D reconstruction of the left clavicle showing a bipolar clavicular displaced fracture.

We report the case of a 78y.o female patient suffering from trauma to the left shoulder due to a car accident with the car tumbling multiple times, X-rays (Fig. 1) and CT scan (Fig. 2) with 3D reconstruction (Fig. 3) done in the ER reveal a bipolar clavicular fracture, the distal third clavicle fracture Allman type II group IIB, and AC joint separation Rockwood type V, group III Allman medial clavicle fracture, on the ipsilateral reverse shoulder prosthesis; as well as 3^rd^ to 7^th^ ribs fracture that were treated conservatively.

A shoulder immobilizer sling was placed initially, and she was scheduled for open reduction and internal fixation of the clavicle fracture.

**Fig. 4:**
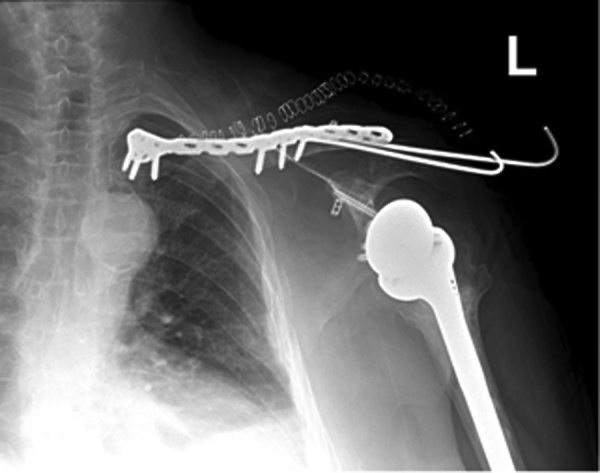
Post operative radiograph of the left shoulder showing plate and screws construct with two K-wires, showing satisfactory alignment of the bipolar clavicular fracture, along with an intact, stable reverse shoulder prosthesis.

Patient was taken to the operating room, under general anesthesia, beach chair position with arm rest, scrubbing and draping done. Using two k-wires under fluoroscopy, reduction and fixation of the lateral part of the fracture to the midshaft of the clavicle. Incision over medial aspect of the clavicle, dissection reaching the bone, reduction and fixation with plate and screws construct. Instability remained over the lateral part so decision to open reduction and internal fixation with fiberwire and endobuttons was taken. Using a small incision over acromioclavicular joint and distal clavicle, reduction and fixation was done with two endobuttons and fiberwire in three planes. Closure was done in the usual fashion, shoulder immobilizer was placed and post operative radiographs were done (Fig. 4). The patient was discharged three days postop.

**Fig. 5:**
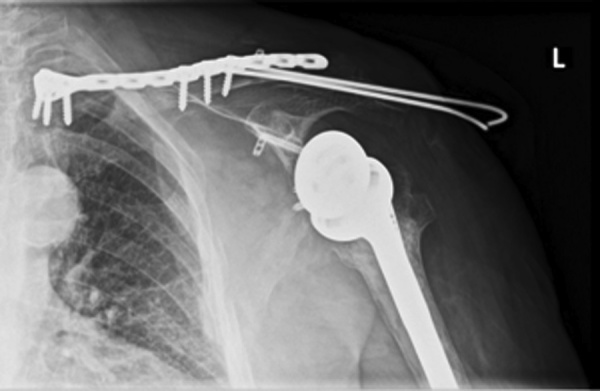
Radiograph of the left shoulder one month post open reduction and internal fixation of bipolar clavicular fracture, showing good bone healing with stable construct and satisfactory alignment, along with an intact reverse shoulder prosthesis.

Postoperatively, the patient was immobilized for 3 weeks then, gentle passive range of motion was initiated for two weeks followed by progressive active range of motion. Follow up radiographs were done at one month post op (Fig. 5).

**Fig. 6:**
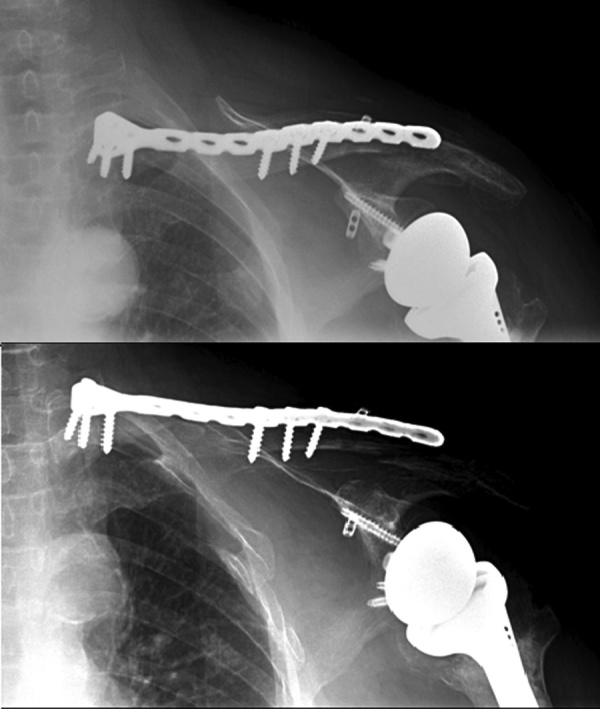
Radiograph of the left shoulder three months post op after removal of the percutaneous K-wires. Good bone healing seen with satisfactory alignment, with stable construct of the previous reverse shoulder prosthesis.

At three months post-op follow up, the percutaneous pins have been removed. The patient recovered most of her range of motion and is pain free, with satisfactory follow up imaging (Fig. 6).

## 3 Discussion

Clavicle fractures are prevalent, accounting for 2.6–4% of all adult fractures and 44% of shoulder girdle injuries. [1,2] Of all clavicle fractures, midshaft fractures are the most common, ranging from 69% to 82%, distal fractures range from 21% to 28%, and proximal fractures range from 2% to 3%. Two incidence peaks have been defined: first, in young men with midshaft fractures due to direct trauma, second, in older women who tend to have fractures associated with osteoporosis during household falls [3]. In older patients, proximal or distal clavicle fractures are more common [1,2]. Segmental clavicle fractures (paired mid-shaft and proximal fracture, or distal fracture mid-shaft fractures) are rare. Only 0.8% of patients had segmental injuries in a study of 614 clavicle fractures [4]. To our knowledge, around 40 bipolar clavicle fracture case reports [5,6], medial and lateral fracture of the clavicle, have been established in the literature [7,8]. Very few have been documented with 3D reconstructed CT scan [9]. It was originally described by Porral in 1831 [10]. It is known as a complete dislocation [11], bipolar dislocation [10], panclavicular dislocation [12], bifocal clavicular dislocation [13], traumatic floating clavicle. [14] It is thought that isolated clavicle fractures result from a direct force on the shoulder tip, most frequently the result of a straightforward collapse or injury to sports. Most clavicle fracture, midshaft and proximal part, are treated conservatively [15]. However, displaced distal clavicle fractures or those medial to the coracoclavicular ligaments require surgical fixation. Segmental fractures are unstable and are at increased risk of nonunion if treated non operatively; therefore, management is by surgical fixation.

Bipolar clavicle injuries are caused by the rotation of the clavicle about its midpoint, leading to posterior dislocation of the AC joint and anterior dislocation of the sternoclavicular joint. If dislocation occurs at both ends, then probably it is due to a major trauma whereby the deforming force acts on the lateral aspect of the shoulder or a powerful force pressing the shoulders together with trunk torsion [16].

Their management is difficult and no consensus exist on the matter. In few cases, it has been treated conservatively [[17], [18], [19]], in other cases open reduction and internal fixation of the distal end [20] while as in other both ends were fixed [21,22]. Fixation can be done with virtually any method including k-wires [23] to plate and screws constructs; however, fixation and reduction must be adequate. Complications include persistence of sternoclavicular joint dislocation with conservative therapy [19], failure of material [23], and non-union.

The increase in detection of these cases may be due to an increase in usage of CT scan imaging which reveals fractures of the medial part of the clavicle not easily visible on a plain X-ray. It points out that in any patient with a clavicle fracture, with a history of sequential forces to the clavicle, a bipolar injury should always be suspected. Our case is special in that it is the first reported, to our knowledge, whereby a bipolar fracture of the clavicle occurs with ribs fractures on the same side of the reverse shoulder prosthesis. We opted for a surgical fixation due to the inherent instability of the fracture in the current situation. Good functional and clinical outcomes were found after multiple physiotherapy sessions. To note, this article has been reported in line with the SCARE 2018 criteria [24].

## 4 Conclusion

This is the first case reported in the literature which shows a bipolar clavicle fracture on an ipsilateral reverse shoulder prosthesis associated with multiple ribs fracture. Surgical treatment was opted due to the nature and pattern of fracture. Post operative follow up showed satisfactory results. No consensus on management exists, but when surgical option is opted for it is best to consider the fracture in its two separate parts and treat each one individually with the construct of choice.

## Funding

No funds were received in support of this study.

## Ethical approval

Ethics committee has given approval for publication.

## Consent

Written informed consent was obtained from the patient for publication of this case report and accompanying images. A copy of the written consent is available for review by the Editor-in-Chief of this journal on request.

No identity identifiers are present whatsoever in the manuscript

## Author contribution

Joseph Maalouly: contributed to the writing and editing of this article.

Dany Aouad: contributed to the writing of this article and the submission process.

Jamal Saade: contributed to the editing of the figures and of the text.

Ghadi Abboud: contributed to the radiological images, and editing of the final text.

Georges El Rassi: contributed with the case, surgical management and editing of the article.

## Registration of research studies

This case has been registered in the IRB committee of St Georges Hospital University Hospital.

## Guarantor

Dr Georges El Rassi.

## Provenance and peer review

Not commissioned, externally peer-reviewed.

## Declaration of Competing Interest

The authors declare no conflict of interest regarding the publication of this article.
